# Effect of acupuncture for disorders of consciousness in patients with stroke: A systematic review and meta-analysis

**DOI:** 10.3389/fneur.2022.930546

**Published:** 2022-10-05

**Authors:** Zhibin Huang, Yuning Chen, Qilan Xiao, Weichuan Kuang, Kun Liu, Ye Jiang, Xi Wen, Weiting Qin, Yue Liu, Tong Liu

**Affiliations:** ^1^Department of Acupuncture and Rehabilitation, The Fifth College of Clinical Medicine, Guangzhou University of Traditional Chinese Medicine, Guangzhou, China; ^2^Department of Rehabilitation, Equivalent Master's Degree Applicants of Guangzhou University of Chinese Medicine, Guangzhou, China; ^3^Department of Rehabilitation, The Sixth Affiliated Hospital of Jinan University, Dongguan, China; ^4^Department of Acupuncture and Rehabilitation, Guangdong Second Hospital of Traditional Chinese Medicine, Guangzhou, China; ^5^Department of Urinary Surgery, The First Affiliated Hospital of Guangzhou University of Traditional Chinese Medicine, Guangzhou, China

**Keywords:** stroke, systematic review, DOC, GCS, GOS, acupuncture

## Abstract

**Background:**

Disorder of consciousness (DOC) is frequent in patients with stroke, which is the second most common cause of death and a leading cause of disability. Acupuncture has been used as a curative method for DOC treatment in China. Nevertheless, no critical systematic review of acupuncture's effect on DOC has been published. This review aims to evaluate the present evidence regarding the efficacy of acupuncture for DOC after stroke.

**Methods:**

Seven databases were searched from their inception to November 1, 2021, containing three English databases (PubMed, Embase, and Cochrane Central Register of Controlled Trials) and four Chinese databases (CNKI, CBM, VIP, and Wanfang Database). The primary outcomes comprise the Glasgow Coma Scale (GCS) and Glasgow Outcome Scale (GOS) before and after treatment. Secondary outcomes involve resuscitation rate, resuscitation time, and adverse events. Data synthesis was calculated by RevMan (V.5.4.1) software. According to the Cochrane Handbook, methodological quality was assessed with the risk of bias tool 2.0 (RoB2).

**Results:**

Seventeen studies containing 1,208 patients were eventually included in our review. Overall, most trials were rated as high or had some concerns regarding the risk of bias. GCS was reported in 16 trials, and a meta-analysis showed that GCS improvement in the acupuncture group was greater than in the non-acupuncture group (MD 1.45, 95% CI 0.94–1.97, *P* < 0.0001). One trial reported that GOS improvement in the acupuncture plus medication group was greater than in the medication group (MD 0.58, 95% CI 0.11–1.05, *P* = 0.01). Another study reported that acupuncture plus medication was statistically more effective in shortening resuscitation time than medication alone (MD−0.89, 95% CI −1.53 to −0.25, *P* = 0.006). Four trials reported that the resuscitation rate in the acupuncture group was higher than without acupuncture intervention (RR 1.68, 95% CI 1.30–2.18, *I*^2^ 0%, *P* = 0.39). Adverse events were reported in two studies, with one case in the acupuncture group suffering from subcutaneous hematoma.

**Conclusion:**

Acupuncture may improve consciousness level, increase the resuscitation rate, and shorten resuscitation time for post-stroke patients with DOC. Adverse events from acupuncture were rare, tolerable, and recoverable. However, the results should be interpreted cautiously, and more rigorous RCTs with better methodology are warranted.

**Systematic review registration:**

https://www.crd.york.ac.uk/PROSPERO/display_record.php?RecordID=289802, identifier 289802.

## Introduction

Stroke, a common acute cerebrovascular accident, can be clinically divided into two types according to pathogenesis: ischemic stroke and hemorrhagic stroke—the former accounts for about 62.4% and the latter, 37.6% (1). High incidence, morbidity, mortality, and recurrence rate constitute the significant characteristics of stroke. From a global perspective, stroke, in particular, has imposed a ponderous burden on developing countries (2). In 2017, the total number of stroke deaths worldwide was 6.16 million, while China accounts for about one-third of the total, with 2.1 million (3). Besides, the overall lifetime risk of stroke in China is 39.9%, the highest in the world, which means that about two out of five people may suffer from a stroke during their lifetime. In 2017, the per capita hospitalization expenses for patients with ischemic and hemorrhagic strokes in China were 9,607 and 18,525 Chinese yuan (CNY), an increase of 60 and 118%, respectively, compared with 2007 (4). Therefore, more attention should be paid to strokes.

Several complications could be involved in stroke, such as consciousness disorder, depression, dysphagia, cognitive impairment, and so on (5). The sequelae of a stroke may lead to a lengthy recovery period, high medical expenses, and a poor prognosis for post-stroke patients (6). Among various sequelae, a disorder of consciousness (DOC) occurs frequently. According to an evidence-based practice guideline for stroke, nearly one out of every three stroke patients suffers from DOC to varying degrees (7). Moreover, it was also shown that 40% of patients with DOC have difficulty regaining normal consciousness (8). In a clinical randomized controlled study involving 6,336 people, the results indicated that the hospital mortality rate of stroke patients with DOC was higher than those without (35.9 vs. 2.6%) (9).

Currently, stroke treatment is based primarily on surgery and drug thrombolytic therapy (10, 11). In terms of stroke sequelae, it has been pointed out that multidisciplinary cooperative rehabilitation units could achieve superior outcomes (12). As a relatively well-recognized traditional therapy, acupuncture has broadly been used in post-stroke rehabilitation in China (13). Studies have indicated that acupuncture treatment for stroke can adjust the stability of the human body environment through the influence of the nerve-endocrine-immune network (14). Specific to stroke, acupuncture affects the neurovascular unit through multiple levels to promote cerebral blood perfusion in the focal area of stroke patients and improve the function of damaged brain cells (15, 16). Furthermore, with respect to the therapeutic method regarding post-stroke patients with impaired consciousness, acupuncture has been carried out in correspondent clinical study (17).

However, no systematic review assessing the effect of acupuncture for DOC has been carried out until now. Consequently, we performed this review to explore whether acupuncture was an efficacious therapy for DOC.

## Method

### Study registration

Our review protocol has been registered on PROSPERO (No. CRD42021289802). The Cochrane Handbook for Systematic Reviews of Interventions and the Preferred Reporting Items for Systematic Reviews and Meta-Analysis Protocol (PRISMA-P) statement guidelines were strictly complied with (18, 19).

### Inclusion criteria for study selection

#### Types of studies

All clinical RCTs were included. Non-randomized or quasi-randomized controlled trials or case reports, case series, systematic reviews and meta-analyses, and animal studies were excluded. For randomized crossover trials, we used the data before crossing.

#### Types of participants

Patients in the trial must be diagnosed with stroke with DOC, such as coma, vegetative state (VS), or minimally conscious state (MCS). The diagnosis was confirmed by an imagelogical examination, clinical signs, and symptoms. The qualified patients were enrolled in the review without regarding any information about their age, sex, race, education, or nationality. Studies focused on DOC due to other causes, such as traumatic brain injury, were excluded.

#### Types of interventions

##### Experimental interventions

Studies that label the intervention “acupuncture,” for example, traditional acupuncture, auricular acupuncture, electro-acupuncture, warm needle, scalp acupuncture, manual acupuncture, fire needle, superficial acupuncture, wrist-ankle acupuncture, and abdominal acupuncture, were included. However, noninvasive methods such as laser acupuncture and point massage were excluded.

##### Control interventions

The control groups could use placebo acupuncture, repetitive transcranial magnetic stimulation (rTMS), hyperbaric oxygen, acupressure, no treatment, non-acupoint acupuncture, medication, massage, etc. Studies that compared acupuncture with another therapy with the same therapy alone were also included. Trials that only involved comparisons between different types of acupuncture were excluded.

### Outcome measures

#### Primary outcomes

The Glasgow Coma Scale (GCS) score and Glasgow Outcome Scale (GOS) score are vital criteria for assessing a conscious state. Hence, the level of consciousness was measured by GCS and GOS before and after treatment. The GCS has three major items, a total of 15 points. The higher the score, the better the consciousness level. The GOS has five points in all; the higher the score, the better the awareness outcome.

#### Secondary outcomes

Resuscitation rate, resuscitation time, and adverse events related to the treatment were extracted from the study as secondary outcomes.

### Search methods for the identification of studies

#### Electronic searches

The following databases were searched from their inception to November 1, 2021, including three English literature databases (PubMed, Embase, and Cochrane Central Register of Controlled Trials) and four Chinese literature databases (Chinese National Knowledge Infrastructure database, Chinese Biomedical database, VIP, and Wanfang Database). Terms of medical subjects (MeSH) and keywords were used individually or in combination during the query. Nonetheless, the search strategy for other databases was slightly modified. Besides, Chinese characters with the same meaning were used for literature retrieval in the Chinese databases. The overall search strategy is displayed in Supplementary Appendix 1.

#### Searching for other resources

Clinical trial registries, dissertations, and gray literature were additionally searched. Furthermore, the reference lists of selected studies were scanned for additional studies. We also searched the WHO International Clinical Trials Registry Platform (ICTRP; http://apps.who.int/trialsearch/), the ClinicalTrials.gov registry (http://clinicaltrials.gov/), the Chinese Clinical Trial Registry, and other relevant trial registries.

#### Data collection and analysis

##### Selection of studies

**Figure 1:**
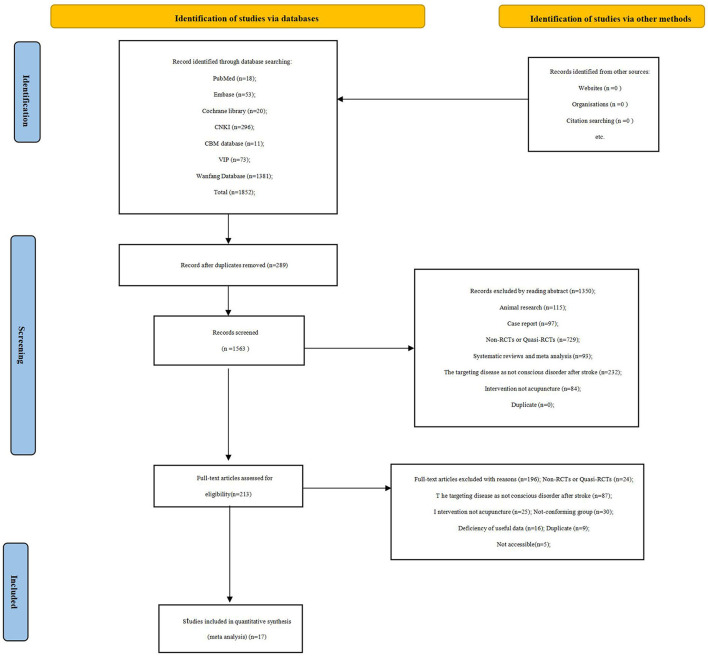
Study flow diagram.

Before the selection of studies, all reviewers received professional training to understand the objective and process of the review. NoteExpress software was applied to upload the studies obtained from electronic databases and other resources. The selection of studies for the current review was performed independently by two reviewers (CYN and HZB) to screen the titles, abstracts, and keywords of all retrieved records separately. Any disagreement was resolved through discussion and adjudication to get a consensus and judged by an arbiter (LT). Details of the selection procedure for studies are shown in a PRISMA flow chart (Figure 1).

#### Data extraction and management

The following key information was extracted from each study: first author, publication year, sample size, characteristics of participants, disease duration, intervention and control, acupoints selected, duration and sessions of treatment, outcome measures, results reported, and adverse events. Study selection was scrutinized independently by two reviewers based on the predetermined inclusion criteria, with disagreement resolved by discussion and adjudication. When the data of articles were insufficient or ambiguous, we tried to contact the corresponding authors for more information.

#### Assessment of the risk of bias in included studies

The research team conducted a risk-of-bias assessment using the risk of bias tool 2.0 (RoB2) from Cochrane. The assessment includes the randomization process, deviations from intended interventions, missing outcome data, measurement of the outcome, and selection of the reported results. The overall judgment of each trial can be “low” or “high” or “some concerns” risk of bias. With any discrepancies resolved through discussion, the research team independently completed the risk-of-bias assessment for each study.

#### Measures of treatment effect

Outcome data were summarized using risk ratio (RR) with 95% confidence intervals (CI) for binary outcomes, while mean difference (MD) with 95% CI for continuous outcomes. WMD with 95% CIs was applied if outcomes were assessed by the same scale, while SMD with 95% CIs was applied if outcomes were assessed by different scales. Revman (V.5.4.1) software was used.

#### Unit of analysis issue

Considering that some studies compared two or more intervention groups with a control group, the research team followed the recommended advice in the Cochrane Handbook version 6.2 (18) and combined groups to create a single pairwise comparison to avoid a unit-of-analysis error.

#### Dealing with missing data

If possible, we tried to contact the corresponding authors by e-mail or telephone to acquire the missing data. When we failed, we analyzed the available data and discussed the potential influence of the missing data in the discussion.

#### Assessment of heterogeneity

We first took the characteristics of patients (e.g., age, sex) and trial design (e.g., adequate sequence generation, blinding of assessors) into account to assess the clinical heterogeneity of the included studies. Then, we used the *I*-squared and Chi^2^ tests to evaluate the statistical heterogeneity of the included studies. A random-effects model was used when *I*^2^ ≥50%, while a fixed-effects model was applied when *I*^2^ < 50%. When significant clinical heterogeneity existed, we used sensitivity analysis and descriptive analysis.

#### Data synthesis and analysis

Review Manager Software (RevMan V.5.4.1) from Cochrane Collaboration was applied for data synthesis and analysis.

#### Assessment of publication bias

If the overall quantity of our study was more than 10, a funnel plot analysis was conducted to determine publication bias.

#### Subgroup analysis

We considered that different add-on treatments to acupuncture might influence the adjunctive effect of acupuncture. We conducted subgroup analysis for primary outcomes as follows: acupuncture plus medication therapy vs. medication therapy alone, acupuncture plus rehabilitation training therapy vs. rehabilitation training therapy alone, acupuncture vs. medication, and acupuncture vs. rehabilitation training therapy.

#### Sensitivity analysis

A sensitivity analysis was performed to identify the robustness of studies according to the comparison between different effect models.

#### Grading the quality of evidence

Grading of Recommendations Assessment, Development, and Evaluation (GRADE) was applied to evaluate the quality of confidence for primary outcomes in included studies (20). The evaluation was divided into four levels: high, moderate, low, or very low.

## Results

### Characteristics of included trials

Through a detailed search of seven electronic databases, a total of 1,852 related articles were obtained. After checking the duplication and reading the titles and abstracts, 213 articles remained. We further read the full text of these papers; a total of 17 studies containing 1,208 patients were eventually included in our systematic review (21–37). A manual review of the references in the included literature did not reveal additional studies of value.

All the included papers were RCTs and published in Chinese journals from 2013 to 2021 in China. The individual sample sizes of the studies ranged from 30 to 114. However, the exact calculation of the sample size was not mentioned in any of the studies. In all included studies, there was no statistically significant difference in the average age of males and females. The treatment course ranged from 7–90 days, and the treatment times of acupuncture ranged from 7–90. Of all the included studies, fourteen studies compared acupuncture plus medication therapy with medication therapy alone (21–35); two studies compared acupuncture plus rehabilitation therapy with rehabilitation therapy (including music therapy and repetitive transcranial magnetic stimulation) alone (36, 37), and three studies compared acupuncture to medication therapy (33–35). One study compared acupuncture to rehabilitation training therapy (repetitive transcranial magnetic stimulation) (37). All the general information is presented in Table 1.

**Table 1:** General information of the included trials.

**First author**	**Year**	**Sample** **Size**	**Age** **(mean ±SD)**	**Sex** **(male/female)**	**Disease** **duration (day)**	**Acu** **retention**	**Regimen**	**Duration of** **treatment**	**Primary** **outcomes**	**Secondary** **outcomes**	**Adverse effects**	**Acupoint selected**
He et al. (21)	2016	T:20	T: 47. 7 ± 11. 4	T: 15/5	T: 1. 5 ± 0. 6	20	T: MT + Acu	20 times,22 days	GCS	resuscitation rate		BaiHui(GV20), SiShenCong(EX-HN1), ShenTing(GV24), Parietal anterior slash, ShuiGou(GV26)
		C: 20	C:45. 6 ± 10. 0	C:13/7	C:1. 7 ± 0. 4		CMT					
Bi et al. (22)	2014	T:38	T:62.33 ± 8.73	T:23/15	T:1.3 ± 0.5	NR	T: MT + Acu	7 times, 7 days	GCS	resuscitation time		NeiGuan(PC6), RenZhong(GV26), SanYingJiao(SP6), JiQuan(HT1), ChiZe(LU5), WeiZhong(BL40)
		C:38	C:60.38 ± 8.26	C:20/18	C:1.2 ± 0.5		C: MT					
WU et al. (23)	2013	T:15	T:52.7 ± 1.3	T:11/4	5	30	T: MT + Acu	30 times,30 days	GCS	resuscitation rate		NeiGuan(PC6), RenZhong(GV26), SanYingJiao(SP6)
		C:15	C:50.1 ± 3.2	C:12/3			C: MT					
Tian et al. (24)	2016	T:50	T:65.61 ± 5.43	NR	≤ 3	30	T: MT + Acu	24 times,30 days	GCS			BaiHui(GV20), NeiGuan(PC6), ChiZe(LU5), WeiZhong(BL40), (ShuiGouGV26), QuChi(LI11), WanGu(GB12), JinJing(BL1), HeGu(LI4), LianQuan(RN23)
		C:50	C:64.18 ± 4.89				C: MT					
Duan et al. (25)	2012	T:20	T:62.20 ± 8.11	T:13/7	≤ 1	30	T: MT + Acu	23 times,30 days	GCS			YiFeng(SJ17), FengChi(GB20), YuYe(EX-HN13), NeiGuan(PC6).
		C:20	C:60.40 ± 7.85	C:14/6			C: MT					RenZhong(GV26), SanYingJiao(SP6), JiQuan(HT1), ChiZe(LU5)
												WeiZhong(BL40), QuChi(LI11)BaiHui(GV20), WanGu(GB12), JinJing(BL1),
Zhao (26)	2017	T:38	T:60.05 ± 10.34	T:22/16	T:2.5 ± 0.8	20	T: MT + Acu	20 times,26 days	GCS, GOS	resuscitation rate	ecchymoma	BaiHui(GV20), SiShenCong(EX-HN1), ShuiGou(GV26), SuLiao(DU25), NeiGuan(PC6)
		C:38	C:59.53 ± 9.51	C:23/15	C:2.3 ± 0.9		C: MT					
OuYang (27)	2018	T:33	T:60.39 ± 13.73	T:19/14	NR	20	T: MT + Acu	20 times, 26 days	GCS		Ecchymoma	BaiHui(GV20), SiShenCong(EX-HN1), TaiYang(EX-HN5), QuBing(GB7), ShuiGou(GV26)
		C:32	C:60.53 ± 9.54	C:20/12			CMT				stuck needle	JianYu(LI15), QuChi(LI11), ZhiGou(SJ6), WaiGuan(SJ5), HeGu(LI4), FuTu(ST32), XueHai(SP10), ZuSanLi(ST36), YangLinQuan(GB34), FengLong(ST40)
Gao (28)	2018	T:32	T:55.72 ± 7.13	T:15/17	T:2.7 ± 0.2	40	T: MT + Acu	24 times,26 days	GCS			ShuiGou(GV26), NeiGuan(PC6), SanYingJiao(SP6)
		C:32	C:63.52 ± 6.41	C:16/16	C:3..5 ± 1.4		C: MT					
Qi (29)	2020	T:40	T: 61.22 ± 11.05	T:25/15	T:45.3 ± 14.7	15	T: MT + Acu	90 times,90 days	GCS			ShuiGou(GV26), BaiHui(GV20), HeGu(LI4), LianQuan(RN23),
		C:40	C:60.84 ± 10.39	C:24/16	C:44.7 ± 15.3		C: MT					TaiChong(LR3), FengChi(GB20), QuBing(GB7), TongLi(HT5)
Huang (30)	2018	T:16	T:57	T:11/5	NR	25	T: MT + Acu	28 times,28 days	GCS			JianYu(LI15), Jianliao(SJ14), JianZhen(SI9), YangXi(LI5), QuChi(LI11),
		C:16	C:54	C:10/6			C: MT					HouXi(SI3), HeGu(LI4), HuanTiao(GB30), FengShi(GB31), ZhongDu(LR6),
												ZuSanLi(ST36), YangLinQuan(GB34), JueGu(GB39)
Liu et al. (31)	2021	T:33	T:63 ± 6	T:19/14	3–7	30	T: MT + Acu	21 times,21 days	GCS	resuscitation rate		YinTang(EX-HN3), ShuiGou(GV26), BaiHui(GV20), ShenTing(GV24), NeiGuan(PC6),
		C:32	C:63 ± 7	C:17/15			C: MT					SanYingJiao(SP6)
Zhang (32)	2015	T:40	T + C:61.2 ± 10.56	T + C:50/30	>0.4	30	T: MT + Acu	45 times, 51 days	GCS			YongQuan(KI1), BaiHui(GV20), SanYingJiao(SP6), ShiXuan(EX-UE11)
		C:40					C: MT					LaoGong(PC8), ShenMen(HT7), NeiGuan(PC6), FengFu(DU16), ShuiGou(GV26)
Wang et al. (33)	2021	T:38	T:65 ± 5	T:21/17	0.4 ± 0.1	30	T: ACU+EDA	10 times, 14 days	GCS			NeiGuan(PC6), SanYingJiao(SP6), ShuiGou(GV26), HeGu(LI4),
		C1:38	C1:64 ± 5	C1:20/18			C1:EDA					WeiZhong(BL40), ChiZe(LU5), JiQuan(HT1)
		C2:38	C2:64 ± 5	C2:22/16			C2:Acu					
Han (34)	2019	T:34	T:49.58 ± 6.52	T:19/15	≤ 1	30	T: ACU+NX	14 times, 14 days	GCS			NeiGuan(PC6), RenZhong(GV26), SanYingJiao(SP6)
		C1:33	C1:49. 72 ± 6. 31	C1:19/14			C1:NX					
		C2:33	C2:48.16 ± 7.24	C1:18/15			C2:Acu					
Yang et al. (35)	2013	T:36	T:58.5 ± 19	T:17/19	T:2.8 ± 0.5	NR	T: ACU+CM	14 times,14 days	GCS			HeGu(LI4), ShuiGou(GV26), LianQuan(RN23), SiShenCong(EX-HN1),
		C1:36	C1: 56.7 ± 21	C1:16/20	C1:2.6 ± 0.7		C1:CM					NeiGuan(PC6), ZuSanLi(ST36), TaiChong(LR3)
		C2:36	C2: 56.8 ± 20	C2:18/18	C2:2.7 ± 0.6		C2:Acu					
Li et al. (36)	2021	T:30	T:53.2 ± 8.2	T: 16/14	T:85 ± 3	30	T:ACU+rTMS	24 times, 28 days	GCS			BaiHui(GV20), RenZhong(GV26), ShenTing(GV24)
		C1:30	C1:53.2 ± 5.2	C1:14/16	C1:85 ± 6		C1:rTMS					NeiGuan(PC6), YongQuan(KI1)
		C2:30	C2:54.5 ± 2.2	C2:15/15	C2:86 ± 2		C2:Acu					
Li et al. (37)	2020	T:24	T:58.03 ± 3.58	T:14/10	3–10	80	T: ACU+HBO	30 times, 30 days	GCS			BaiHui(GV20), QianDing(DU21), HouDing(DU19), RenZhong(GV26), ShenShu(BL23)
		C:24	C:57.11 ± 3.61	C:13/11			C: HBO					XinShu(BL15), TaiXi(KI3), TaiChong(LR3), ShenMen(HT7), TongLi(HT5)

Acu, acupuncture; MT, medication therapy; RT, rehabilitation therapy; EDA, Edaravone; NX, Naloxone; CM, yiqixingshen fan; rTMS, repetitive transcranial magnetic stimulation; HBO, hyperbaric oxygen therapy; NR, not referred; Year, publication year; w, week; T, treatment group; C, control group; GCS, Glasgow Coma Scale; GOS, Glasgow Outcome Scale.

### Risk of bias within the trial

**Figure 2:**
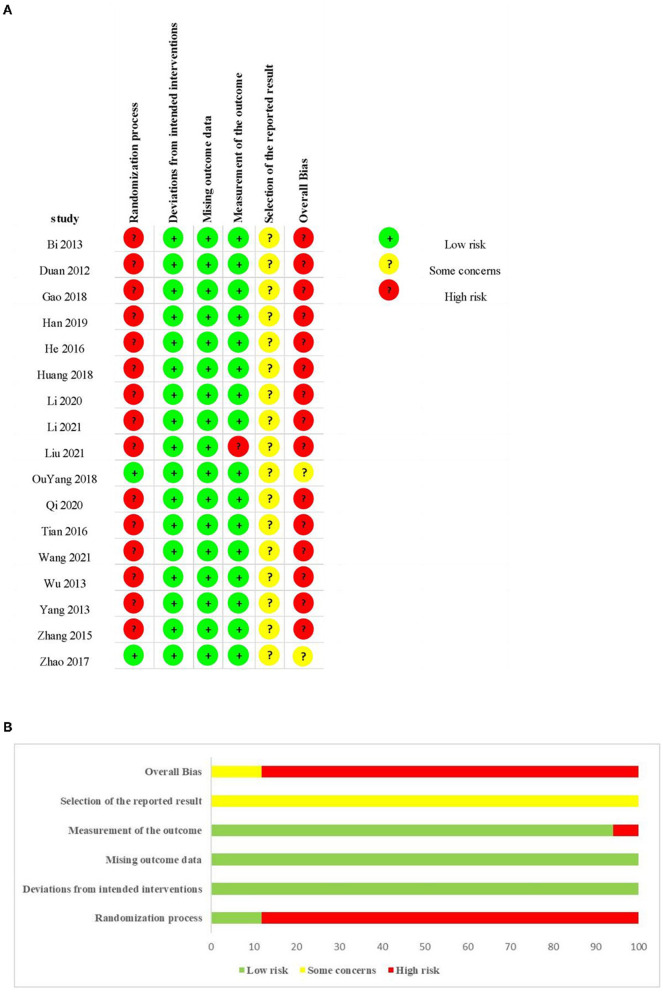
Assessment of risk of bias (ROB2) using the Cochrane tool. **(A)** ROB graph and **(B)** ROB summary.

Two (11.8%) studies (26, 27) were rated as having a low risk of bias for the randomization process because they provided a detailed random sequence generation process, and the allocation sequence was concealed. Fifteen trials (88.2%) (21–25, 28–37) were rated as having a high risk of bias for the randomization process because none provided a specific randomization method or random sequence allocation concealment. Regarding deviations from intended interventions and missing outcome data parts, all studies could be assessed as having a low risk of bias according to the descriptions in the method and results in sections of the included trials. As for the measurement of the outcome part, one study (5.8%) (31) had a high risk of bias because it measured the primary outcome in part of the population. In the evaluation of a selection of the reported results, all of the studies could be rated as having some level of concern about bias because of the absence of protocols of included trial. As for the overall bias, most of the trials (88.2%) (21–25, 28–37) were rated as having a high risk of bias, while the remaining two trials (11.8%) (26, 27) were rated as having some level of concerns about bias. The specific information is presented in Figure 2.

### Outcomes of meta-analysis

#### The Glasgow Coma Scale

**Figure 3:**
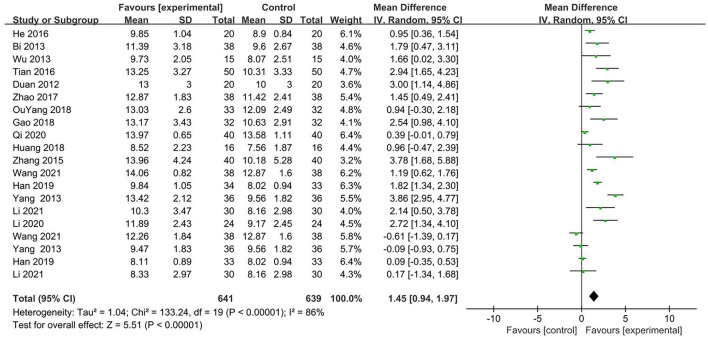
Forest plot and meta-analysis of GCS (Glasgow coma scale).

The GCS was reported in 16 articles (94.1%) (21–30, 32–37). Because of the considerable heterogeneity of the included studies, we used a random-effects model for the corresponding analysis. Results showed that the advancement of GCS scores in the acupuncture group was better than that in the non-acupuncture group (MD 1.45, 95% CI 0.94–1.97, *P* < 0.0001; Figure 3).

#### Glasgow Outcome Scale

Moreover, only one article reported GOS as the primary outcome (26). The experimental group applied acupuncture plus medication therapy, while the control group used medication therapy. The result indicated that the improvement of GOS scores in the acupuncture plus medication group was greater than in the medication group (MD 0.58, 95% CI 0.11 to 1.05, *P* = 0.01).

### Resuscitation rate

**Figure 4:**
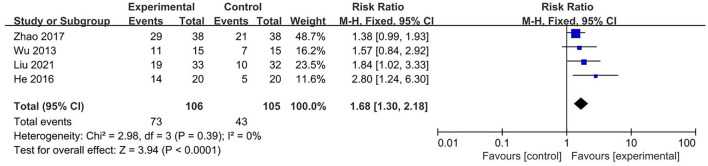
Forest plot and meta-analysis of resuscitation rate.

Four studies reported the resuscitation rate as an indicator (21, 23, 26, 31). Included studies showed no significant heterogeneity, and the fix-effects model was used for the analysis. The result showed that the resuscitation rate was substantially higher in the acupuncture plus medication group than in the medication group (RR 1.68, 95% CI 1.30–2.18, I2 0%, *P* = 0.39; Figure 4).

### Resuscitation time

Only one study reported resuscitation time (22). Results showed that acupuncture in conjunction with medication could significantly shorten resuscitation time than medication alone (MD −0.89, 95% CI −1.53 to −0.25, *P* = 0.006).

### Adverse events

Two studies reported adverse events were reported in two studies (26, 27) (11.8%) reported adverse events. Both trials reported one case in the acupuncture group suffering from subcutaneous hematoma, respectively. Fortunately, the symptoms were obviously alleviated after general treatment.

### Subgroup analysis

#### Acupuncture in conjunction with medication vs. medication

**Figure 5:**
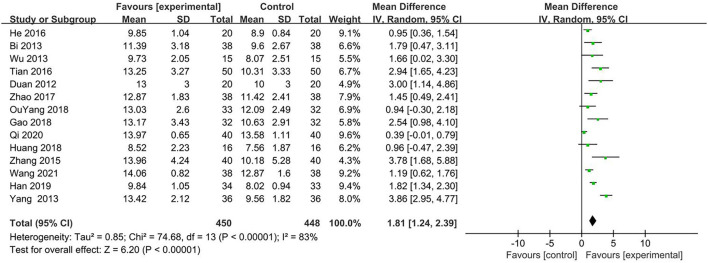
Forest plot of GCS (Glasgow coma scale) comparing acupuncture plus MT (medication therapy) vs. MT (medication therapy) alone.

Fifteen (21–35) trials compared acupuncture plus medication with medication by GCS. However, one trial (31) only evaluated the GCS score of the awakened patients. So 14 trials (82.35%) (21–30, 32–35) with 898 participants were included in the final analysis. A random effects model assessed the effects due to significant heterogeneity. Results showed that GCS in the acupuncture plus medication group was significantly higher than in the medication group (MD 1.81, 95% CI 1.24–2.39, *P* < 0.0001; Figure 5).

#### Acupuncture in conjunction with rehabilitation vs. rehabilitation

**Figure 6:**
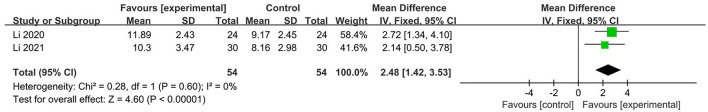
Forest plot of GCS (Glasgow coma scale) comparing acupuncture plus RT (rehabilitation therapy) vs. RT (rehabilitation therapy) alone.

Two (11.76%) (36, 37) trials with 108 participants compared acupuncture plus rehabilitation with rehabilitation by GCS. Considering the minor heterogeneity, we applied the fixed effects model to analyze the effects. The result showed that GCS in the acupuncture plus rehabilitation group was significantly higher than in the rehabilitation group (MD 2.48, 95% CI 1.42–3.53, *P* < 0.0001; Figure 6).

#### Acupuncture vs. medication

**Figure 7:**
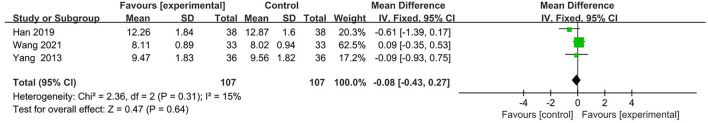
Forest plot of GCS (Glasgow coma scale) comparing acupuncture vs. MT (medication therapy) alone.

Three trials (17.65%) (33–35) with 214 participants compared acupuncture with medication by GCS. The fixed effects model was used because no obvious heterogeneity was found. The result showed no significant difference between the acupuncture and medication groups (MD −0.08, 95% CI −0.43 to 0.27, *P* = 0.64) (Figure 7).

#### Acupuncture vs. rehabilitation

One trial (5.88%) (37) with 60 participants compared acupuncture with rehabilitation by GCS. Results showed no significant difference between the acupuncture and rehabilitation groups (MD 0.17, 95% CI −1.34 to 1.68, *P* = 0.82).

### Sensitivity analysis

Different effect models may affect the analysis of the results; therefore, we tested the stability of the meta-analysis by comparing the difference between the fixed-effects model and the random-effects model. It showed that there were no significant differences between the two statistical models. The detailed results are shown in Table 2.

**Table 2:** Results of the sensitivity analysis.

**Study type**	**Study quantity**	**Participants**		**Study heterogeneity**				**Analysis model**	**MD (95% CI)**	***P*-value**
		**Experiment group**	**Control group**	**chi^2^**	***df***	***I*^2^,%**	***P*-value**			
**GCS**										
Acu plus MT vs. MT	14	450	448	74.68	13	0.83	<0.0001	Random	1.81 (1.24, 2.39)	<0.0001
								Fixed	1.34 (1.13, 1.55)	<0.0001
Acu plus RT vs. RT	2	54	54	0.28	1	0	0.60	Random	2.48 (1.42, 3.53)	<0.0001
								Fixed	2.48 (1.42, 3.53)	<0.0001
Acu vs. MT	3	107	107	2.36	2	0.15	0.31	Random	−0.11 (−0.51, 0.29)	0.60
								Fixed	−0.08 (−0.43, 0.27)	0.64
Acu vs. RT	1	30	30	*	*	*	*	Random	0.17 (−1.34, 1.68)	0.82
								Fixed	0.17 (−1.34, 1.68)	0.82
**Resuscitation rate**										
Acu plus RT / RT	4	106	105	1.38	3	0	0.71	Random	3.33 (1.86, 5.97)	<0.0001
								Fixed	3.34 (1.87, 5.95)	<0.0001

Acu, acupuncture; MT, medication therapy; RT, rehabilitation therapy; CI, confidence interval; df, degrees of freedom; MD, mean difference; GCS, Glasgow Coma Scale.

^*^Insufficient data.

### Quality of evidence

We used the Grading of Recommendations Assessment, Development, and Evaluation (GRADE) system to evaluate the quality of confidence for included studies. The results generally showed a very low level of evidence concerned with the fact that acupuncture can improve GCS, resuscitation rate, and resuscitation time in post-stroke patients with DOC. Although the level of evidence is moderate regarding the effect of acupuncture in promoting GOS, this result is limited by the small sample size. Specific information is listed in Table 3.

**Table 3:** Summary of findings and strength of evidence for outcomes.

Acupuncture for disorders of consciousness in patients with stroke						
Patient or population: patients with the conscious disorder after stroke Settings:: Hospitals in mainland China Intervention: Acupuncture						
**Outcomes**	Illustrative comparative risks*(95% CI)		**Relative effect (95% CI)**	**No of Participants (studies)**	**Quality of the evidence (GRADE)**	**Comments**
	**Assumed risk**	**Corresponding risk**				
	**Control**	**GCS**				
**GCS**		**The mean gcs in the intervention groups was One higher (0.82 to 1.17 higher)**		**1,280 (16 studies)**	**⊕⊖⊖⊖** **very low1,2,3**	
**GCS - Acu plus MT vs. MT**		**The mean gcs - acu plus mt vs. mt in the intervention groups was 1.34 higher (1.13 to 1.55 higher)**		**898 (14 studies)**	**⊕⊖⊖⊖** **very low1,2,3**	
**GCS - Acu plus RT vs. RT**		**The mean gcs - acu plus rt vs. rt in the intervention groups was 2.48 higher (1.42 to 3.53 higher)**		**108 (2 studies)**	**⊕⊖⊖⊖** **very low1,2,3**	
**GCS - Acu vs. MT**		**The mean gcs - acu vs. mt in the intervention groups was 0.08 lower (0.43 lower to 0.27 higher)**		**214 (3 studies)**	**⊕⊖⊖⊖** **very low1,2,3,4**	
**GCS - Acu vs. RT**		**The mean gcs - acu vs. rt in the intervention groups was 0.17 higher (1.34 lower to 1.68 higher)**		**60 (1 study)**	**⊕⊖⊖⊖** **very low1,2,4**	
**GOS**		**The mean gos in the intervention groups was 0.58 higher (0.11 to 1.05 higher)**		**76 (1 study)**	**⊕⊖⊖⊖** **moderate3**	
**Resuscitation rate**		**Study population**	**OR 3.34 (1.87 to 5.95)**	**211 (4 studies)**	**⊕⊖⊖⊖** **very low1,2,3**	
	**410 per 1,000**	**698 per 1,000 (565 to 805)**				
	**Moderate**				
	**390 per 1,000**	**681 per 1,000 (545 to 792)**				
**Resuscitation time**		**The mean resuscitation time in the intervention groups was 0.89 lower (1.53 to 0.25 lower)**		**76 (1 study)**	**⊕⊖⊖⊖** **very low1,2,3**	

^*^The basis for the assumed risk (e.g., the median control group risk across studies) is provided in footnotes. The corresponding risk (and its 95% confidence interval) is based on the assumed risk in the comparison group and the relative effect of the intervention (and its 95% CI).

CI, confidence interval; OR, odds ratio.

GRADE Working Group grades of evidence. High quality: Further research is unlikely to change our confidence in the effect estimate. Moderate quality: Further research is likely to have an important impact on our confidence in the effect estimate and may change the estimate. Low quality: Further research is very likely to have an important impact on our confidence in the effect estimate and is likely to change the estimate. Very low quality: We are very uncertain about the estimate.

1 The overall quality of rob is low. 2Two fail to provide the study protocol, and the study design and data collection have obvious shortcomings. 3 see the result of publication Figure 5. 4 NO so related to the study found.

### Publication bias

**Figure 8:**
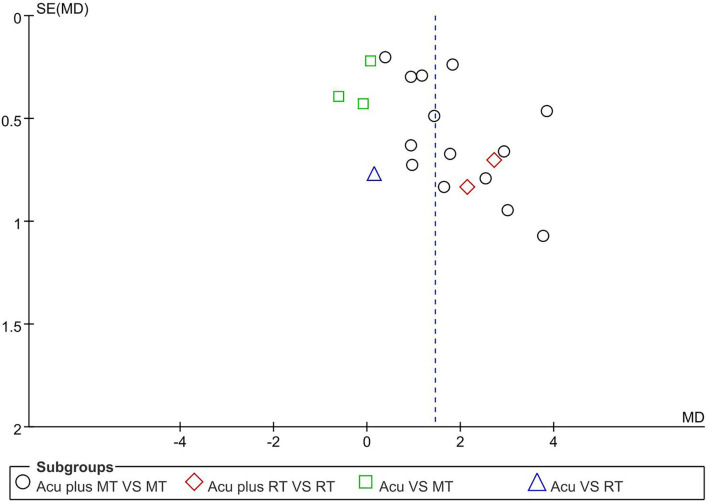
Funnel plot of GCS (Glasgow coma scale).

Publication bias was evaluated with a funnel plot. The results showed that the distribution of the funnel plot failed to be symmetrical, and the lower part of the graph was vacant. According to the results, included studies may have potential publication bias (Figure 8).

## Discussion

In our systematic review, 17 RCTs involving 1,208 patients were eventually included for meta-analysis. Results showed that acupuncture might be more effective in improving consciousness, increasing resuscitation rate, and shortening resuscitation time than those patients without acupuncture intervention. According to our subgroup analysis, compared to medication and rehabilitation alone, acupuncture plus medication and acupuncture plus rehabilitation had more advantages in improving GCS for stroke patients with DOC. Additionally, two studies reported acupuncture-related adverse events, which were mild subcutaneous hematoma and obviously alleviated after general treatment. The results of sensitivity analysis showed that the effect of acupuncture was relatively stable, but the findings in our study had been influenced by methodological flaws and should be considered with caution.

It is worth mentioning that our results suggest that acupuncture combined with drugs and rehabilitation therapy is far more effective than drugs and rehabilitation alone. There may be two reasons for this: one is the superposition of the therapeutic effects of acupuncture itself, and another may be the possible synergistic effect between acupuncture and other therapies. A study by Xia et al. (38) explored the synergistic effects of electro-acupuncture and bone mesenchymal stem cell transplantation on repairing thin endometrial injuries in rats and found that electro-acupuncture promotes the paracrine effect of BMSCs at the site of local injury and improves the ability of BMSCs to differentiate into cells required for tissue regeneration. Another study by Zhou et al. (39) discussed the synergistic and attenuated effects of electro-acupuncture for aconitine in the treatment of heart failure and concluded that electro-acupuncture achieves the synergism/attenuation effect of aconitine for the improvements in heart failure probably by upregulating the expression of SERCA2a and downregulating the expression of PLB. The research team led by Zhou et al. (40) found that acupuncture can significantly improve the respiratory function of asthmatics and increase metallothionein-2 (MT-2) protein content, which was validated as a new target for bronchial asthma. All these findings suggest that acupuncture might represent a good alternative or complementary treatment to conventional management in clinical settings by the same or unique mechanism, which is worth further discussion and exploration.

Several acupoints could be applied in acupuncture manipulation, as shown in Table 1. In all the included studies, the most commonly used acupoints were Shuigou acupoint (GV26), which was used in 16 studies; Neiguan acupoint (PC6), used in 12 studies; Sanyinjiao acupoint (SP6), used in nine studies; and Baihui acupoint (GV20), used in 10 studies. Several studies have revealed that acupuncture could achieve satisfactory treatment effects on stroke, especially for DOC (41, 42). Najem's study (43) indicated that acupuncture could reduce the efficacy of secondary brain injuries by reducing systemic and local inflammation, oxidative stress, intracellular calcium overload, neuronal regeneration, and growth factor release. Tang's research also revealed that acupuncture could activate genes modulating neuronal projections (P2rx7, P2rx3, Trpv1, Tacr1, and Cacna1d), protein secretion (Exoc1, Exoc3l1, Fgb, and Fgr), and dopamine (DA) receptor D3 (Drd3) in the ventral periaqueductal gray (vPAG), as well as the expression and excitability of DA and P2RX7 neurons (44). These may be the potential mechanisms of acupuncture for consciousness improvement in patients following stroke.

Previous systematic reviews have paid attention to the effect of acupuncture on disorders of consciousness after traumatic brain injury (45, 46). Their pooled analyses indicated that acupuncture might have a superior effect on GCS score, GOS score, efficacy rate, ADL, and mortality. However, no review has reported the efficacy of acupuncture on consciousness disorders after stroke. To the best of our knowledge, this meta-analysis of 17 clinical types of research is the first systematic review to address this topic. Therefore, this is a big step forward. We rigorously carried out this meta-analysis according to the Cochrane Collaboration and PRISMA guidelines. Eligible RCTs in seven electronic databases were extensively searched. As the primary outcome measures, GCS and GOS scores have been widely used in the clinic to assess consciousness in stroke patients. However, only one article applied GOS to evaluate the prognosis of the patient, which was a pity. Despite its simplicity, GOS is still the most widely used instrument for measuring the outcome of DOC (47, 48). Therefore, future research is required to focus on this indicator to better observe the outcome of patients and provide more comprehensive data for future meta-analysis. To some extent, our findings provide evidence for the clinical application of acupuncture in stroke patients with DOC.

However, there are still some limitations in our research. Our results were encouraging but not convincing because most trials had a high risk of bias, which may have caused an overestimation or underestimation of the true treatment effect. Firstly, all the included 17 articles were published in China, which may affect the generality of the results. Secondly, the result of the funnel graph also showed a potential publication bias; this could be that most of the included articles were of positive results, and articles with negative results were more difficult to publish. Then, some research results may be against the interests of the funder, forced to be stranded, and cannot be published. Thirdly, the number of studies included was extremely limited, with only 17 papers included in the total, with a maximum sample size of only 114. The treatment duration, frequency of acupuncture, and acupoint selection also varied a lot. Fourthly, allocation concealment and blinding methods were ignored in most studies, with only two (26, 27) studies specifying the allocation concealment and blinding method. Fifthly, despite subgroup analysis, there is still heterogeneity in some comparisons, such as Figure 5, which compared acupuncture plus medication with medication by GCS. The reason may be different drugs or different selected acupoints, and we hope that as more and more studies appear, more accurate analyses and conclusions can be drawn. All the above factors may lead to large heterogeneity, which limits the reliability of the results. Thus, to obtain a more definitive answer to the question of the efficacy of acupuncture for DOC, the field needs more carefully designed and conducted trials. Researchers should use adequate randomization methods and ensure that the group assignment is adequately concealed. Those techniques are both critical to avoiding systematic differences between the baseline characteristics of the compared groups. Because it is almost impossible for a therapist to be blinded to the acupuncture intervention, it may be important to blind the participants and the outcome assessor.

## Conclusion

Acupuncture may be more effective in improving consciousness level, increasing the resuscitation rate, and shortening resuscitation time than in patients without acupuncture intervention. Adverse events from acupuncture were rare, tolerable, and recoverable. However, the results should be interpreted cautiously, and more rigorous RCTs with better methodology are warranted in the area.

## Data availability statement

The original contributions presented in the study are included in the article/Supplementary material, further inquiries can be directed to the corresponding author.

## Author contributions

TL was responsible for the study's design, supervision, and manuscript revision. ZH and YC contributed equally by both drafting the manuscript and undertaking the trial registration. QX, WK, KL, YJ, XW, WQ, and YL were involved in the interpretation of the study findings. All authors commented on early drafts and approved the final version of the manuscript.

## Funding

This study was financially supported by the National Science Foundation of China (No. 82174482), the Foundation of Guangzhou Municipal Science and Technology Bureau (No. 202102020510), the Special Research of the Traditional Chinese Medicine Bureau of Guangdong Province (No. 20203001), and the Natural Science Foundation Project of Guangdong Province (No. 2022A1515011676).

## Conflict of interest

The authors declare that the research was conducted in the absence of any commercial or financial relationships that could be construed as a potential conflict of interest.

## Publisher's note

All claims expressed in this article are solely those of the authors and do not necessarily represent those of their affiliated organizations, or those of the publisher, the editors and the reviewers. Any product that may be evaluated in this article, or claim that may be made by its manufacturer, is not guaranteed or endorsed by the publisher.
