# Supplementary material for: Effect of acupuncture for disorders of consciousness in patients with stroke: A systematic review and meta-analysis
Source: Front Neurol. 2022 Oct 5;13:930546. doi: 10.3389/fneur.2022.930546 (PMC9581330; doi:10.3389/fneur.2022.930546)
Supplement: Supplementary file 1 [file Table_1.DOCX]

Appendix 1：search in databases

**CNKI**

( ( ( ( ( ( ( ( ( 主题%='昏迷' or 题名%='昏迷' or title=xls('昏迷') or v_subject=xls('昏迷') ) OR ( 主题%='意识障碍' or 题名%='意识障碍' or title=xls('意识障碍') or v_subject=xls('意识障碍') ) ) OR ( 主题%='中脏腑' or 题名%='中脏腑' or title=xls('中脏腑') or v_subject=xls('中脏腑') ) ) OR ( 主题%='最小意识状态' or 题名%='最小意识状态' or title=xls('最小意识状态') or v_subject=xls('最小意识状态') ) ) OR ( 主题%='植物状态' or 题名%='植物状态' or title=xls('植物状态') or v_subject=xls('植物状态') ) ) OR ( 主题%='无反应觉醒综合征' or 题名%='无反应觉醒综合征' or title=xls('无反应觉醒综合征') or v_subject=xls('无反应觉醒综合征') ) ) OR ( 主题%='促醒' or 题名%='促醒' or title=xls('促醒') or v_subject=xls('促醒') ) ) AND ( ( ( ( ( ( ( ( ( 主题%='中风' or 题名%='中风' or title=xls('中风') or v_subject=xls('中风') ) OR ( 主题%='卒中' or 题名%='卒中' or title=xls('卒中') or v_subject=xls('卒中') ) ) OR ( 主题%='脑血管意外' or 题名%='脑血管意外' or title=xls('脑血管意外') or v_subject=xls('脑血管意外') ) ) OR ( 主题%='脑梗死' or 题名%='脑梗死' or title=xls('脑梗死') or v_subject=xls('脑梗死') ) ) OR ( 主题%='脑缺血' or 题名%='脑缺血' or title=xls('脑缺血') or v_subject=xls('脑缺血') ) ) OR ( 主题%='脑梗塞' or 题名%='脑梗塞' or title=xls('脑梗塞') or v_subject=xls('脑梗塞') ) ) OR ( 主题%='脑出血' or 题名%='脑出血' or title=xls('脑出血') or v_subject=xls('脑出血') ) ) OR ( 主题%='脑溢血' or 题名%='脑溢血' or title=xls('脑溢血') or v_subject=xls('脑溢血') ) ) OR ( 主题%='脑栓塞' or 题名%='脑栓塞' or title=xls('脑栓塞') or v_subject=xls('脑栓塞') ) ) ) AND ( ( ( ( ( ( ( ( ( ( ( ( ( ( ( ( ( ( ( ( 主题%='浮针' or 题名%='浮针' or title=xls('浮针') or v_subject=xls('浮针') ) OR ( 主题%='温针' or 题名%='温针' or title=xls('温针') or v_subject=xls('温针') ) ) OR ( 主题%='腹针' or 题名%='腹针' or title=xls('腹针') or v_subject=xls('腹针') ) ) OR ( 主题%='飞针' or 题名%='飞针' or title=xls('飞针') or v_subject=xls('飞针') ) ) OR ( 主题%='靳三针' or 题名%='靳三针' or title=xls('靳三针') or v_subject=xls('靳三针') ) ) OR ( 主题%='蜂针' or 题名%='蜂针' or title=xls('蜂针') or v_subject=xls('蜂针') ) ) OR ( 主题%='芒针' or 题名%='芒针' or title=xls('芒针') or v_subject=xls('芒针') ) ) OR ( 主题%='毫针' or 题名%='毫针' or title=xls('毫针') or v_subject=xls('毫针') ) ) OR ( 主题%='针法' or 题名%='针法' or title=xls('针法') or v_subject=xls('针法') ) ) OR ( 主题%='醒脑开窍' or 题名%='醒脑开窍' or title=xls('醒脑开窍') or v_subject=xls('醒脑开窍') ) ) OR ( 主题%='针灸' or 题名%='针灸' or title=xls('针灸') or v_subject=xls('针灸') ) OR ( 主题%='针刺' or 题名%='针刺' or title=xls('针刺') or v_subject=xls('针刺') ) ) OR ( 主题%='电针' or 题名%='电针' or title=xls('电针') or v_subject=xls('电针') ) ) OR ( 主题%='手针' or 题名%='手针' or title=xls('手针') or v_subject=xls('手针') ) ) OR ( 主题%='耳针' or 题名%='耳针' or title=xls('耳针') or v_subject=xls('耳针') ) ) OR ( 主题%='头皮针' or 题名%='头皮针' or title=xls('头皮针') or v_subject=xls('头皮针') ) ) OR ( 主题%='火针' or 题名%='火针' or title=xls('火针') or v_subject=xls('火针') ) ) OR ( 主题%='皮内针' or 题名%='皮内针' or title=xls('皮内针') or v_subject=xls('皮内针') ) ) OR ( 主题%='三棱针' or 题名%='三棱针' or title=xls('三棱针') or v_subject=xls('三棱针') ) ) OR ( 主题%='腕踝针' or 题名%='腕踝针' or title=xls('腕踝针') or v_subject=xls('腕踝针') ) ) ) )

**WANFANG**

主题：（中风 or 卒中 or 脑血管意外 or 脑梗死 or 脑缺血 or 脑梗塞 or 脑出血 or 脑溢血 or 脑栓塞） and 主题：（针刺 or 电针 or 手针 or 耳针 or 头皮针 or 火针 or 皮内针 or 三棱针 or 腕踝针 or 浮针 or 温针 or 腹针 or 飞针 or 靳三针 or 蜂针 or 芒针 or 毫针 or 针法 or 醒脑开窍） and 主题：（昏迷 or 意识障碍 or 中脏腑 or 最小意识状态 or 植物状态 or 无反应觉醒综合征 or 促醒）

**VIP**

[((((((((((题名或关键词=中风 OR 题名或关键词=卒中) OR 题名或关键词=脑血管意外) OR 题名或关键词=脑梗死) OR 题名或关键词=脑缺血) OR 题名或关键词=脑梗塞) OR 题名或关键词=脑出血) OR 题名或关键词=脑溢血) OR 题名或关键词=脑栓塞) AND (((((((((((((((((((题名或关键词=针灸 OR 题名或关键词=针刺) OR 题名或关键词=电针) OR 题名或关键词=手针) OR 题名或关键词=耳针) OR 题名或关键词=头皮针) OR 题名或关键词=火针) OR 题名或关键词=皮内针) OR 题名或关键词=三棱针) OR 题名或关键词=腕踝针) OR 题名或关键词=浮针) OR 题名或关键词=温针) OR 题名或关键词=腹针) OR 题名或关键词=飞针) OR 题名或关键词=靳三针) OR 题名或关键词=蜂针) OR 题名或关键词=芒针) OR 题名或关键词=毫针) OR 题名或关键词=针法) OR 题名或关键词=醒脑开窍)) AND ((((((题名或关键词=昏迷 OR 题名或关键词=意识障碍) OR 题名或关键词=中脏腑) OR 题名或关键词=最小意识状态) OR 题名或关键词=植物状态) OR 题名或关键词=无反应觉醒综合征) OR 题名或关键词=促醒))](http://qikan.cqvip.com/Qikan/search/index?LngMySearHistoryIdGuid=7428dd4f-fa7f-4ead-a5af-971b6475f7c5&from=Qikan_Article_History)

**CBM**

[("针灸"[加权:扩展] OR "针刺"[加权:扩展] OR "电针"[加权:扩展] OR "手针"[加权:扩展] OR "耳针"[加权:扩展] OR "头皮针"[加权:扩展] OR "火针"[加权:扩展] OR "皮内针"[加权:扩展] OR "三棱针"[加权:扩展] OR "腕踝针"[加权:扩展] OR "浮针"[加权:扩展] OR "温针"[加权:扩展] OR "腹针"[加权:扩展] OR "飞针"[加权:扩展] OR "靳三针"[加权:扩展] OR "蜂针"[加权:扩展] OR "芒针"[加权:扩展] OR "毫针"[加权:扩展] OR "针法"[加权:扩展] OR "醒脑开窍"[加权:扩展]) AND ("昏迷"[加权:扩展] OR "意识障碍"[加权:扩展] OR "中脏腑"[加权:扩展] OR "最小意识状态"[加权:扩展] OR "植物状态"[加权:扩展] OR "无反应觉醒综合征"[加权:扩展] OR "促醒"[加权:扩展]) AND ("中风"[加权:扩展] OR "卒中"[加权:扩展] OR "脑血管意外"[加权:扩展] OR "脑梗死"[加权:扩展] OR "脑缺血"[加权:扩展] OR "脑梗塞"[加权:扩展] OR "脑出血"[加权:扩展] OR "脑溢血"[加权:扩展] OR "脑栓塞"[加权:扩展] OR "脑血管病"[加权:扩展])](javascript:toDoRelimitSearch();)

**PUBMED**

1. cerebrovascular disorders[mh]
2. Cerebrovascular Disease*[tiab] OR Intracranial Disorder*[tiab] OR Cerebrovascular Disorder*[tiab] OR Intracranial Disease*[tiab] OR Brain Vascular Disorder*[tiab] OR Vascular Disorder*, Brain[tiab] OR Vascular Disorder*, Intracranial[tiab] OR Vascular Disease*, Intracranial[tiab] OR Intracranial Vascular Disease*[tiab] OR Disease*, Cerebrovascular[tiab] OR Vascular Disorder*, Intracranial[tiab] OR Cerebrovascular Insufficienc*[tiab] OR Cerebrovascular Occlusion*[tiab] OR Insufficienc*, Cerebrovascular[tiab] OR Occlusion*, Cerebrovascular[tiab]
3. Arter* Disease*, Carotid[tiab] OR Arter* Disorder*, Carotid[tiab] OR Disorder*, Carotid Artery[tiab] OR Carotid Arter* Disorder*[tiab] OR Carotid Arter* Disease*[tiab] OR Arterial Disease*, External Carotid[tiab] OR External Carotid Artery Disease*[tiab] OR Atherosclerotic Disease*, Carotid[tiab] OR Carotid Atherosclerotic Disease*[tiab] OR Carotid Atheroscleros*[tiab] OR Arter* Disease*, Common Carotid[tiab] OR Internal Carotid Arter* Disease*[tiab] OR Arter* Disease*, Internal Carotid[tiab]
4. Arter* Brain Disorder*[tiab] OR Arter* Brain Disease*[tiab] OR Arter* Disease*, Brain[tiab] OR Brain Arter* Disorder*[tiab] OR Intracranial Arter* Disease*[tiab] OR Brain Arter* Disease*[tiab] OR Intracranial Arter* Disorder*[tiab] OR Brain Disorder*, Arter*[tiab] OR Brain Disease*, Arter*[tiab] OR Arter* Brain Disease[tiab] OR Arter* Disease*, Intracranial[tiab] OR Arter* Disorder*, Intracranial[tiab]
5. Posterior Fossa Hemorrhage*[tiab] OR Cerebrum Hemorrhage*[tiab] OR Brain Hemorrhage*[tiab] OR Intracranial Hemorrhage*[tiab] OR Cerebral Hemorrhage*[tiab] OR Parenchymal Hemorrhage*[tiab] OR Intracerebral Hemorrhage*[tiab] OR Ganglionic Hemorrhage*[tiab] OR Putamen Hemorrhage*[tiab] OR Hemorrhage*, Posterior Fossa[tiab] OR Hemorrhage*, Cerebrum[tiab] OR Hemorrhage*, Cerebral[tiab] OR Hemorrhage*, Brain[tiab] OR Hemorrhage*, Intracranial[tiab] OR Hemorrhage*, Intracerebral[tiab] OR Hemorrhage*, Cerebrum[tiab] OR Hemorrhage*, Basal Gangli*[tiab] OR Hematoma*, Basal Gangli*[tiab] OR Hemorrhage, Putam*[tiab] OR Putaminal Hematoma*[tiab]
6. Cerebral Ischemia*[tiab] OR Brain Ischemia*[tiab] OR Ischemia*, Cerebral[tiab] OR Ischemic Encephalopath*[tiab] OR Encephalopath*, Ischemic[tiab] OR Ischemia*, Brain[tiab] OR Brain Infarct*[tiab] OR Infarct*, Brain[tiab] OR Infarction*, Posterior Circulation, Brain[tiab] OR Anterior Cerebral Circulation Infarction*[tiab] OR Infarction*, Anterior Cerebral Circulation[tiab] OR Infarction*, Anterior Circulation, Brain[tiab]
7. Benedict Syndrome*[tiab] OR Claude Syndrome*[tiab] OR Foville Syndrome*[tiab] OR Weber Syndrome*[tiab] OR Millard-Gublar Syndrome*[tiab] OR Millard-Gublar Syndrome*[tiab] OR Top of the Basilar Syndrome*[tiab] OR Dorsolateral Medullary Syndrome*[tiab] OR Lateral Bulbar[tiab] OR Wallenberg Syndrome*[tiab] OR Lateral Medullary Syndrome*[tiab] OR Posterior Inferior Cerebellar Artery Syndrome*[tiab] OR Wallenberg's Syndrome*[tiab] OR Wallenbergs Syndrome*[tiab] OR Infarction*, Brainstem[tiab] OR Brainstem Infarction*[tiab] OR Stem Infarct*, Brain[tiab] OR Infarct*, Brain Stem[tiab] OR Brain Stem Infarct*[tiab] OR Brainstem Stroke[tiab] OR Stroke, Brainstem[tiab] OR Syndrome, Millard-Gublar[tiab] OR Syndrome*, Lateral Medullary[tiab] OR Syndrome*, Wallenberg[tiab] OR Medullary Syndrome*, Dorsolateral[tiab]

#8 Transient Ischemic Attack*[tiab] OR Transient Cerebral Ischemia*[tiab] OR Attack*, Transient Ischemic[tiab] OR Ischemic Attack*, Transient[tiab] OR Cerebral Ischemia*, Transient[tiab] OR Ischemia*, Transient Cerebral[tiab] OR Transient Brainstem Ischemia*[tiab] OR Brainstem Ischemia*, Transient[tiab] OR Ischemia*, Transient Brainstem[tiab] OR Brain Stem Ischemia, Transient[tiab]

#9 Vertebral Artery Insufficienc*[tiab] OR Vertebral Artery Ischemia*[tiab] OR Vertebral Artery Stenos*[tiab] OR Basilar Artery Insufficienc*[tiab] OR Basilar Artery Ischemia*[tiab] OR Basilar Artery Stenos*[tiab] OR Insufficienc*, BasilarArtery[tiab] OR Insufficienc*, Basilar[tiab] OR Artery Ischemia*, Basilar[tiab]

OR Ischemia*, Basilar Artery[tiab] OR Basilar Insufficienc*[tiab] OR Stenos*,Basilar Artery[tiab] OR Artery Stenos*, Basilar[tiab] OR Insufficienc*, Vertebral Artery[tiab] OR Artery Ischemia*, Vertebral[tiab] OR Artery Insufficienc*, Vertebral[tiab] OR Ischemia*, Vertebral Artery[tiab] OR Vertebro-Basilar Ischemia*[tiab] OR Vertebro-Basilar Insufficienc*[tiab] OR Vertebro-Basilar Dolichoectasia*[tiab] OR Vertebrobasilar Ischemia*[tiab] OR Vertebrobasilar Insufficienc*[tiab] OR Vertebrobasilar Dolichoectasia*[tiab] OR Vertebro Basilar Ischemia*[tiab] OR Vertebro Basilar Insufficienc*[tiab] OR Vertebro Basilar Dolichoectasia*[tiab] OR Ischemia*, Vertebro-Basilar[tiab] OR Insufficienc*, Vertebro-Basilar[tiab] OR Insufficienc*, Vertebrobasilar[tiab] OR Ischemia*, Vertebrobasilar[tiab] OR Ischemia, Vertebrobasilar[tiab] OR Ischemia*, Vertebro-Basilar[tiab] OR Dolichoectasia*, Vertebrobasilar[tiab] OR Vertebrobasilar Dolichoectasia*[tiab] OR Stenos*, Vertebral Artery[tiab] OR Artery Stenos*, Vertebral[tiab] OR "Cerebral Embolism and Thrombosis"[tiab]

#10 Apoplexy[tiab] OR Acute Stroke*[tiab] OR Cerebral Stroke*[tiab] OR Cerebrovascular Stroke*[tiab] OR Cerebrovascular Accident*[tiab] OR Brain Vascular Accident*[tiab] OR Cerebrovascular Apoplexy[tiab] OR Cerebrovascular Accident*, Acute[tiab] OR Vascular Accident*, Brain[tiab] OR Stroke*, Cerebrovascular[tiab] OR Stroke*, Acute[tiab] OR Stroke*, Cerebral[tiab]

#11 #1 OR #2 OR #3 OR #4 OR #5 OR #6 OR #7 OR #8 OR #9 OR #10

#12 acupuncture[MeSH Terms]

#13 (Electroacupuncture)[Title/Abstract] OR (Auricular acupuncture)[Title/Abstract]

OR (Fire needle)[Title/Abstract] OR (Warm needle)[Title/Abstract] OR (Manual

acupuncture)[Title/Abstract] OR (Scalp acupuncture)[Title/Abstract])

#14 #12 OR #13

#15 consciousness disorder[MeSH Terms]

#16 (Consciousness Disorder)[Title/Abstract] OR (Consciousness, Level Depressed)[Title/Abstract] OR (Depressed Level of Consciousness)[Title/Abstract] OR (Semiconsciousness)[Title/Abstract] OR (Consciousness, Level Altered)[Title/Abstract] OR (Altered Level of Consciousness)[Title/Abstract] OR (coma)[Title/Abstract] OR (vegetative state)[Title/Abstract] OR (minimally conscious state)[Title/Abstract] OR (late emergence from minimally conscious state)[Title/Abstract] OR (unresponsive wakefulness syndrome)[Title/Abstract]

#17 #15 OR #16

#18 #11 AND #14 AND #17

**Embase**

1. 'cerebrovascular disease'/exp
2. 'brain angiopathy':ti,ab OR 'brain circulation failure':ti,ab OR 'brain vascular disease$':ti,ab OR 'cerebral small vessel disease$':ti,ab OR ('cerebral vascular':ti,ab AND (disease:ti,ab OR disorder:ti,ab OR disturbance:ti,ab OR lesion:ti,ab)) OR ((brain:ti,ab OR cerebral:ti,ab) AND vasculopathy:ti,ab) OR (cerebrovascular:ti,ab AND (damage:ti,ab OR disorder$:ti,ab OR lesion:ti,ab OR pathology:ti,ab OR syndrome:ti,ab))
3. 'basal gangli* cerebrovascular disease':ti,ab OR 'basal gangli* h$emorrhage':ti,ab OR 'cerebrovascular disease, basal gangli*':ti,ab OR 'haemorrhage, basal gangli*':ti,ab OR 'hemorrhage, basal gangli*':ti,ab OR 'h$emorrhage, putamen':ti,ab OR 'putam* h$ematoma':ti,ab OR 'putam* h$emorrhage':ti,ab OR (carotid:ti,ab AND arterial:ti,ab AND (disease$:ti,ab OR disorder$:ti,ab)) OR (carotid:ti,ab AND (arteriopathy:ti,ab OR disease:ti,ab)) OR (carotid:ti,ab AND artery:ti,ab AND (disease$:ti,ab OR disorder$:ti,ab)) OR ((cerebral:ti,ab OR intracranial:ti,ab) AND embolism:ti,ab AND thrombosis:ti,ab) OR 'carotid artery atherosclerosis':ti,ab OR ('carotid atherosclerotic':ti,ab AND (disease$:ti,ab OR plaque$:ti,ab))
4. 'artery occlusion, carotid':ti,ab OR ('carotid artery':ti,ab AND ((constriction:ti,ab OR occlusion:ti,ab OR occlusive:ti,ab) AND disease:ti,ab OR stenosis:ti,ab)) OR (carotid:ti,ab AND (obliteration:ti,ab OR occlusion:ti,ab OR stenosis:ti,ab)) OR 'arterial thrombosis, carotid':ti,ab OR 'carotid artery thrombus':ti,ab OR 'carotid thrombosis':ti,ab OR 'thrombosis, carotid artery':ti,ab OR 'artery occlusion, internal carotid':ti,ab OR 'internal carotid arterial occlusion':ti,ab OR 'internal carotid occlusion':ti,ab
5. (brain:ti,ab OR cerebral:ti,ab OR interhemispheric:ti,ab OR intracerebral:ti,ab OR intracranial:ti,ab) AND h$ematoma:ti,ab OR 'h$ematoma, brain':ti,ab OR 'bleeding, corpus callosum':ti,ab OR ((brain:ti,ab OR cerebral:ti,ab OR intracerebral:ti,ab OR intracortical:ti,ab OR intracranial:ti,ab OR periventricular:ti,ab) AND (bleeding:ti,ab OR h$emorrhage$:ti,ab OR microh$emorrhage$:ti,ab OR microbleed:ti,ab)) OR ('corpus callosum':ti,ab AND (bleeding:ti,ab OR h$emorrhage:ti,ab)) OR 'encephalorrhagia':ti,ab OR (h$emorrhage,:ti,ab AND (brain:ti,ab OR intracranial:ti,ab)) OR (h$emorrhagic:ti,ab AND (apoplexy:ti,ab OR stroke:ti,ab)) OR (h$emorrhagic:ti,ab AND stroke:ti,ab AND intracerebral:ti,ab AND bleeding:ti,ab) OR 'hematencephalon':ti,ab OR 'intracranial h$emorrhage':ti,ab OR 'posterior fossa h$emorrhage':ti,ab OR ((brain:ti,ab OR cerebral:ti,ab OR cerebrovascular:ti,ab OR cortical:ti,ab OR hemispher*:ti,ab) AND infarc*:ti,ab) OR 'infarction, brain':ti,ab
6. 'anterior cerebral artery infarction':ti,ab OR 'artery disease$, cerebral':ti,ab OR 'cerebral artery disease$':ti,ab OR (infarction,:ti,ab AND (anterior:ti,ab OR middle:ti,ab OR posterior:ti,ab) AND cerebral:ti,ab AND artery:ti,ab) OR 'intracranial arterial disease$':ti,ab OR ((middle:ti,ab OR posterior:ti,ab) AND cerebral:ti,ab AND artery:ti,ab AND infarction:ti,ab) OR 'brain stem infarctions':ti,ab OR 'brainstem infarction':ti,ab OR 'infarction, brain stem or cerebellar infarction':ti,ab
7. ('accident, cerebrovascular':ti,ab OR 'acute cerebrovascular lesion':ti,ab OR 'acute focal cerebral vasculopathy':ti,ab OR ((acute:ti,ab OR apoplectic:ti,ab OR cryptogenic:ti,ab) AND stroke:ti,ab) OR 'apoplex*':ti,ab OR 'blood flow disturbance, brain':ti,ab OR (brain:ti,ab AND (accident:ti,ab OR attack:ti,ab OR 'blood flow disturbance':ti,ab OR insul*:ti,ab OR 'isch$emic attack':ti,ab OR 'vascular accident':ti,ab)) OR (cerebral:ti,ab AND (apoplexia:ti,ab OR insul*:ti,ab OR stroke:ti,ab OR (vascular:ti,ab AND (accident:ti,ab OR insufficiency:ti,ab)))) OR 'cerebro vascular accident':ti,ab OR (cerebrovascular:ti,ab AND (arrest:ti,ab OR failure:ti,ab OR injury:ti,ab OR insufficiency:ti,ab OR insul*:ti,ab)) OR 'cerebrum vascular accident':ti,ab OR 'cva':ti,ab OR (isch$emic:ti,ab AND ('cerebral attack':ti,ab OR seizure:ti,ab)) OR 'stroke':ti,ab OR 'embolic stroke':ti,ab OR 'lacunar infarction':ti,ab OR 'stroke, lacunar':ti,ab OR 'acute isch$emic stroke':ti,ab OR (brain:ti,ab AND 'arterial insufficiency':ti,ab) OR 'circulation disorder':ti,ab OR 'brain ischaemia':ti,ab OR ('cerebral blood':ti,ab AND ('circulation disorder':ti,ab OR 'flow disorder':ti,ab)) OR (cerebral:ti,ab AND (circulation:ti,ab OR circulatory:ti,ab) AND disorder:ti,ab) OR ((cerebral:ti,ab OR cerebrovascular:ti,ab OR neural:ti,ab) AND isch$emia:ti,ab) OR 'cerebrovascular circulation disorder$':ti,ab OR 'chronic isch$emic stroke':ti,ab OR (isch$emi?:ti,ab AND cerebri:ti,ab) OR 'brain disease$':ti,ab OR encephalopathy:ti,ab OR stroke:ti,ab OR ((cerebral:ti,ab OR intracranial:ti,ab) AND vasospasm:ti,ab)) AND vasospasm,:ti,ab AND (brain:ti,ab OR cerebral:ti,ab OR intracranial:ti,ab)
8. 'anoxic isch$emic':ti,ab AND encephalopathy:ti,ab OR 'brain hypoxi? isch$emia':ti,ab OR 'brain hypoxia-isch$emia':ti,ab OR ('brain hypoxic isch$emic':ti,ab AND (damage:ti,ab OR injur*:ti,ab)) OR 'brain isch$emia hypoxia':ti,ab OR 'brain isch$emia-hypoxia':ti,ab OR 'cerebral hypoxi? isch$emia':ti,ab OR ('cerebral hypoxic isch$emic':ti,ab AND (damage:ti,ab OR injur*:ti,ab)) OR 'cerebral isch$emia hypoxia':ti,ab OR 'hie (hypoxic isch$emic encephalopathy)':ti,ab OR 'hypoxia-isch$emia, brain':ti,ab OR ('hypoxic isch$emic':ti,ab AND (brain:ti,ab OR cerebral:ti,ab) AND (damage:ti,ab OR injur*:ti,ab)) OR 'hypoxic isch$emic encephalopathy':ti,ab
9. 'brain transient isch$emic attack':ti,ab OR 'cerebral isch$emia, transient':ti,ab OR 'circulatory epilepsy':ti,ab OR 'epilepsy circulatory':ti,ab OR 'isch$emic attack':ti,ab OR 'tia':ti,ab OR (transient:ti,ab AND (brain:ti,ab OR cerebral:ti,ab) AND isch$emia:ti,ab) OR ('transient isch$emic':ti,ab AND (attack:ti,ab OR seizure:ti,ab)) OR 'brain blood vessel malformation':ti,ab OR ((brain:ti,ab OR cerebral:ti,ab OR cerebrum:ti,ab) AND vascular:ti,ab AND (anomaly:ti,ab OR malformation:ti,ab)) OR 'cerebrovascular anomaly':ti,ab OR 'vascular malformation, cerebrum':ti,ab
10. 'aneurysm, intracranial':ti,ab OR 'arteriovenous aneurysm, brain':ti,ab OR 'arteriovenous cerebral aneurysm':ti,ab OR 'arteriovenous fistula, brain':ti,ab OR 'brain aneurysm, arteriovenous':ti,ab OR ('brain arteriovenous':ti,ab AND (aneurysm:ti,ab OR fistula:ti,ab OR shunt:ti,ab)) OR 'cerebral aneurysm, arteriovenous':ti,ab OR ('cerebral arteriovenous':ti,ab AND (fistula:ti,ab OR malformation$:ti,ab)) OR 'intracranial arteriovenous malformation$':ti,ab
11. 'brain artery':ti,ab AND (obstruction:ti,ab OR occlusion:ti,ab OR thrombosis:ti,ab) OR (brain:ti,ab AND (thromboembolism:ti,ab OR thrombosis:ti,ab)) OR 'brain vascular obstruction':ti,ab OR ('cerebral artery':ti,ab AND (occlusion:ti,ab OR thrombosis:ti,ab)) OR 'cerebral thrombosis':ti,ab OR 'cerebrovascular disease, occlusive':ti,ab OR (cerebrovascular:ti,ab AND (obliteration:ti,ab OR obstruction:ti,ab OR occlusion:ti,ab)) OR 'cerebrovascular occlusi* disease':ti,ab OR 'cerebrovascular thrombosis':ti,ab OR 'intracranial artery thrombosis':ti,ab OR 'intracranial thrombosis':ti,ab OR 'thromboembolism, brain':ti,ab OR (thrombosis:ti,ab AND (cerebri:ti,ab OR intracranial:ti,ab)) OR 'thrombosis, brain artery':ti,ab
12. medial:ti,ab AND (brain:ti,ab OR cerebral:ti,ab) AND 'artery occlusion':ti,ab OR 'middle cerebral artery stenosis':ti,ab OR 'arteria vertebralis occlusion':ti,ab OR ('vertebral artery':ti,ab AND (obstruction:ti,ab OR occlusion:ti,ab)) OR 'vertebral occlusion':ti,ab OR ('lateral medullary':ti,ab AND (disease:ti,ab OR syndrome:ti,ab)) OR 'wallenberg syndrome':ti,ab
13. #1 OR #2 OR #3 OR #4 OR #5 OR #6 OR #7 OR #8 OR #9 OR #10 OR #11 OR #12
14. 'acupuncture'/exp
15. electroacupuncture:ab,ti OR 'auricular acupuncture':ab,ti OR 'fire needle':ab,ti OR 'warm acupuncture':ab,ti OR 'manual acupuncture':ab,ti OR 'scalp acupuncture':ab,ti
16. #14 OR #15
17. 'consciousness disorder'/exp
18. 'consciousness,level depressed':ab,ti OR 'depressed level of consciousness':ab,ti OR semiconsciousness:ab,ti OR 'consciousness, level altered':ab,ti OR 'altered level of consciousness':ab,ti OR coma:ab,ti OR 'vegetative state':ab,ti OR 'minimally conscious state':ab,ti OR 'late emergence from minimally conscious state':ab,ti OR 'unresponsive wakefulness syndrome':ab,ti
19. #17 OR #18
20. #13 AND #16 AND #19

**Cochrane Library**

1. MeSH descriptor: [Cerebrovascular Disorders] explode all trees
2. (((Cerebrovascular OR Intracranial) (Disease? OR Disorder?)) OR Brain Vascular Disorder? OR Vascular Disorder?, Brain OR Vascular Disorder?, Intracranial OR Vascular Disease?, Intracranial OR Intracranial Vascular Disease? OR Disease?, Cerebrovascular OR Vascular Disorder?, Intracranial OR (Cerebrovascular (Insufficienc* OR Occlusion?)) OR Insufficienc*, Cerebrovascular OR Occlusion?, Cerebrovascular):ti,ab,kw (Word variations have been searched)
3. (Arter* Disease?, Carotid OR Arter* Disorder?, Carotid OR Disorder?, Carotid Artery OR Carotid Arter* Disorder? OR Carotid Arter* Disease? OR Arterial Disease?, External Carotid OR External Carotid Artery Disease? OR Atherosclerotic Disease?, Carotid OR Carotid Atherosclerotic Disease? OR Carotid Atheroscleros* OR Arter* Disease?, Common Carotid OR Internal Carotid Arter* Disease? OR Arter* Disease?, Internal Carotid):ti,ab,kw (Word variations have been searched)
4. ((Arter* Brain (Disorder? OR Disease?)) OR Arter* Disease?, Brain OR ((Brain OR Intracranial) Arter* (Disorder? OR Disease?)) OR Brain Disorder?, Arter* OR Brain Disease?, Arter* OR Arter* Brain Disease OR Arter* Disease?, Intracranial OR Arter* Disorder?, Intracranial):ti,ab,kw (Word variations have been searched) 155650
5. (((Posterior Fossa OR Cerebrum OR Brain OR Intracranial OR Cerebral OR Parenchymal OR Intracerebral OR Ganglionic OR Putamen) Hemorrhage?) OR Hemorrhage?, Posterior Fossa OR Hemorrhage?, Cerebrum OR Hemorrhage?, Cerebral OR Hemorrhage?, Brain OR Hemorrhage?, Intracranial OR Hemorrhage?, Intracerebral OR Hemorrhage?, Cerebrum OR Hemorrhage?, Basal Gangli* OR Hematoma?, Basal Gangli* OR Hemorrhage, Putam* OR Putaminal Hematoma?):ti,ab,kw (Word variations have been searched)
6. (((Cerebral OR Brain) Ischemia?) OR Ischemia?, Cerebral OR Ischemic Encephalopath* OR Encephalopath*, Ischemic OR Ischemia?, Brain OR Brain Infarct* OR Infarct*, Brain OR Infarction?, Posterior Circulation, Brain OR Anterior Cerebral Circulation Infarction? OR Infarction?, Anterior Cerebral Circulation OR Infarction?, Anterior Circulation, Brain):ti,ab,kw (Word variations have been searched)
7. (((Benedict OR Claude OR Foville OR Weber OR Millard-Gublar OR Millard-Gublar OR Top of the Basilar OR Dorsolateral Medullary OR Vieseaux-Wallenberg OR Lateral Bulbar OR Wallenberg OR Lateral Medullary OR Posterior Inferior Cerebellar Artery OR Vieseaux Wallenberg OR Wallenberg's OR Wallenbergs) Syndrome?) OR Infarction?, Brainstem OR Brainstem Infarction? OR Stem Infarct*, Brain OR Infarct*, Brain Stem OR Brain Stem Infarct* OR Brainstem Stroke OR Stroke, Brainstem OR Syndrome, Millard-Gublar OR Syndrome?, Lateral Medullary OR Syndrom?, Vieseaux-Wallenberg OR Syndrome?, Wallenberg OR Medullary Syndrome?, Dorsolateral):ti,ab,kw (Word variations have been searched)
8. (((Cerebral OR Brain) (Ischemia Hypoxia? OR Hypoxia-Ischemia? OR Ischemia-Hypoxia? OR Hypoxia Ischemia? OR Anoxia-Ischemia? OR Ischemia-Anoxia? OR Anoxia Ischemia? OR Ischemia Anoxia?)) OR Ischemia-Hypoxia?, Cerebral OR Hypoxia Ischemia?, Cerebral OR Ischemia Hypoxia?, Cerebral OR Hypoxia-Ischemia?, Cerebral OR Ischemia-Hypoxia?, Brain OR Ischemia Hypoxia?, Brain OR Hypoxia-Ischemias, Brain OR Hypoxia Ischemia, Brain OR Ischemia Anoxia?, Cerebral OR Anoxia-Ischemia?, Cerebral OR Anoxia Ischemia?, Cerebral OR Ischemia-Anoxia, Cerebral OR Anoxia-Ischemia?, Brain OR Ischemia-Anoxia?, Brain OR Anoxia Ischemia?, Brain OR Ischemia Anoxia?, Brain OR ((Anoxic-Ischemic OR Anoxic Ischemic OR Hypoxic-Ischemic OR Ischemic-Hypoxic OR Hypoxic Ischemic OR Ischemic Hypoxic) Encephalopath*) OR Encephalopath*, Anoxic Ischemic OR Encephalopath*, Anoxic-Ischemic OR Encephalopath*, Ischemic-Hypoxic OR Encephalopath*, Hypoxic Ischemic OR Encephalopath*, Hypoxic-Ischemic):ti,ab,kw (Word variations have been searched)
9. (((Vertebral OR Basilar) Artery (Insufficienc* OR Ischemia? OR Stenos?s)) OR Insufficienc*, Basilar Artery OR Insufficienc*, Basilar OR Artery Ischemia?, Basilar OR Ischemia?, Basilar Artery OR Basilar Insufficienc* OR Stenos?s, Basilar Artery OR Artery Stenos?s, Basilar OR Insufficienc*, Vertebral Artery OR Artery Ischemia?, Vertebral OR Artery Insufficienc*, Vertebral OR Ischemia?, Vertebral Artery OR ((Vertebro-Basilar OR Vertebrobasilar OR Vertebro Basilar) (Ischemia? OR Insufficienc* OR Dolichoectasia?)) OR Ischemia?, Vertebro-Basilar OR Insufficienc?, Vertebro-Basilar OR Insufficienc?, Vertebrobasilar OR Ischemia?, Vertebrobasilar OR Ischemia, Vertebrobasilar OR Ischemia?, Vertebro-Basilar OR Dolichoectasia?, Vertebrobasilar OR Vertebrobasilar Dolichoectasia? OR Stenos?s, Vertebral Artery OR Artery Stenos?s, Vertebral):ti,ab,kw (Word variations have been searched) 53067
10. ("Cerebral Embolism and Thrombosis" OR "Embolism and Thrombosis, Brain" OR "Brain Embolism and Thrombosis"):ti,ab,kw (Word variations have been searched)
11. (Apoplexy OR ((Acute OR Cerebral OR Cerebrovascular) Stroke?) OR ((Cerebrovascular OR Brain Vascular) Accident?) OR Cerebrovascular Apoplexy OR Cerebrovascular Accident?, Acute OR Vascular Accident?, Brain OR Stroke?, Cerebrovascular OR Stroke?, Acute OR Stroke?, Cerebral):ti,ab,kw (Word variations have been searched)
12. #1 OR #2 OR #3 OR #4 OR #5 OR #6 OR #7 OR #8 OR #9 OR #10 OR #11 831958
13. (Consciousness Disorder):ti,ab,kw OR (Consciousness, Level Depressed):ti,ab,kw OR
14. (Depressed Level of Consciousness):ti,ab,kw OR (Semiconsciousness):ti,ab,kw OR (Consciousness, Level Altered):ti,ab,kw
15. (Altered Level of Consciousness):ti,ab,kw OR (coma):ti,ab,kw OR (vegetative state):ti,ab,kw OR (minimally conscious state):ti,ab,kw OR (late emergence from minimally conscious state):ti,ab,kw
16. (unresponsive wakefulness syndrome):ti,ab,kw
17. #14 OR #15 OR #16
18. #13 OR #17
19. MeSH descriptor: [Acupuncture] explode all trees
20. (Electroacupuncture OR Auricular acupuncture OR Fire needle OR Warm needle OR Manual acupuncture OR Scalp acupuncture):ti,ab,kw
21. #19 OR #20
22. #21 AND #18 AND #12
